# BTEX-K Ameliorates Rheumatoid Arthritis Through Regulating the NF-κB and PPAR-γ Signaling Pathways in Incomplete Freund’s Adjuvant-Induced Arthritis Mice

**DOI:** 10.3390/biomedicines13071524

**Published:** 2025-06-22

**Authors:** Joonpyo Hong, Jin-Ho Lee, Ga Young Lee, Jin-Hwan Oh, Hana Lee, Han Sung Kim, Tack-Joong Kim

**Affiliations:** 1 Division of Biological Science and Technology, Yonsei University, Wonju 26493, Republic of Korea; hhjoonpyo@naver.com (J.H.); drlogos@naver.com (J.-H.L.); 2 Central Research Institute, BTGin Co., Ltd., Deajeon 34024, Republic of Korea; gylee6409@gmail.com (G.Y.L.); jimhwano@naver.com (J.-H.O.); 3 Department of Biomedical Engineering, Yonsei University, Wonju 26493, Republic of Korea; hanah4378@naver.com (H.L.); hanskim@yonsei.ac.kr (H.S.K.); 4 Research & Development Center, Doctor TJ Co., Ltd., Wonju 26493, Republic of Korea

**Keywords:** dried red ginseng, BTEX-K, pro-inflammatory cytokines, nitric oxide, MAPK pathway, chronic inflammation

## Abstract

**Background/Objectives**: Degenerative arthritis is a chronic inflammatory disease marked by tissue degradation and vascular fibrosis. Macrophages play a central role in the inflammatory response by releasing mediators such as nitric oxide (NO), interleukin (IL)-6, tumor necrosis factor alpha (TNF-α), and prostaglandin E2 (PGE2). This study aimed to investigate the anti-inflammatory potential of BTEX-K, a formulation of dried red ginseng combined with alpha-galactosidase, in lipopolysaccharide (LPS)-stimulated cells. **Methods**: LPS-treated immune cells were used to assess the anti-inflammatory effects of BTEX-K. The levels of NO, IL-6, TNF-α, and PGE2 were measured following BTEX-K treatment. The protein expression of inducible nitric oxide synthase (iNOS) and cyclooxygenase-2 (COX-2) was evaluated. Cytotoxicity assays were conducted to determine whether the observed effects were due to cell viability loss. The involvement of MAPK signaling and NF-κB pathway modulation was examined by analyzing JNK phosphorylation, IκB degradation, and PPAR-γ expression. **Results**: BTEX-K significantly reduced the production of NO, IL-6, TNF-α, and PGE2 in LPS-treated cells without inducing cytotoxicity. The protein expression levels of iNOS and COX-2 were also suppressed. Furthermore, BTEX-K inhibited the LPS-induced phosphorylation of JNK in the MAPK pathway. It restored IκB levels and suppressed NF-κB activation by preventing the downregulation of PPAR-γ. **Conclusions**: BTEX-K demonstrates notable anti-inflammatory effects by inhibiting key inflammatory mediators and signaling pathways in immune cells. These findings support its therapeutic potential in mitigating inflammation-related symptoms, including pain, swelling, and redness, commonly seen in degenerative arthritis.

## 1. Introduction

Rheumatoid arthritis (RA) is a chronic autoimmune disease that primarily affects the synovial membrane, leading to persistent joint inflammation. It often starts in small joints, such as those of the fingers and wrists, and can progress to involve multiple joints and organs, causing cartilage damage, bone erosion, and joint deformity. RA affects approximately 0.5–1.0% of the global population and greatly reduces the quality of life [1]. Although the exact cause is unclear, RA is believed to result from an abnormal immune response that drives chronic inflammation and tissue destruction.

An inflammatory response is a defense mechanism stimulated by physical or chemical damage to tissues or a bacterial infection [2]. In chronic inflammation, the inflammatory reaction and healing process occur simultaneously. Although the inflammatory response is an essential function, long-term inflammatory reactions caused by the uncontrolled or excessive production of inflammatory mediators are harmful and are involved in the development of many diseases, including arthritis, diabetes, cancer, cardiovascular disease, Alzheimer’s disease, atherosclerosis, multiple sclerosis, and nerve damage [3]. Macrophages are pivotal innate immune cells that are involved in the primary defense against pathogen invasion and play an important role in phagocytosis, sterilization, the removal of damaged tissue and aging cells, tissue development and recovery, and antigen information delivery [4,5].

LPS is a well-known endotoxin and an essential structural component in the outer membrane of Gram-negative bacteria and is one of the most potent microbial initiators of inflammation. LPS interacts with Toll-like receptor 4 (TLR 4) to activate the macrophages involved in the infection response, leading to the production of various molecules and pro-inflammatory cytokines, such as interleukin (IL)-6 and tumor necrosis factor alpha (TNF-α) [6]. Nitric oxide (NO) has also been implicated as a mediator of inflammatory response. LPS-stimulated macrophages produce large amounts of NO over a long period of time [7]. COX-2 is released from immune cells, such as macrophages and lubricating membrane cells, in response to infection, damage, or other stress and produces large amounts of prostaglandins (PGs) that sensitize pain receptors and induce inflammatory states [8].

TLR 4 is a major LPS receptor. Combination with LPS results in the activation of downstream mediators, including the transcription factor nuclear factor (NF-κB), which increases the production of infectious molecules, such as cytokines [9]. Both the TLR 4 and MAPK signaling pathways are associated with inflammation [10]. TLR 4 can also activate the MyD88-dependent NF-κB and MAPK pathways, leading to ERK, JNK, and p38 MAPK activation, which can induce the release of different levels of inflammatory cytokines [11].

IL-6 and TNF-α are pro-inflammatory cytokines that are released from macrophages upon stimulation with LPSs [12]. TNF-α is involved in a wide range of inflammatory, infectious, autoimmune, and malignant conditions and released in response to infection. TNF-α overexpression can cause chronic inflammatory and autoimmune diseases, such as chronic inflammatory arthritis, inflammatory bowel disease (IBD), and multiple sclerosis (MS) [13]. Prostaglandin E2 (PGE2) is involved in tissue damage by inducing the production of matrix metalloproteinases in inflammatory diseases, and large amounts of PGE2 are produced in macrophages obtained from patients with rheumatoid arthritis [14].

NO generated by iNOS in an inflammatory state is known to promote not only inflammatory reactions, such as vascular permeability and edema, but also the biosynthesis of inflammatory mediators to deepen inflammation [15]. COX-2 is an inducible enzyme that is induced by inflammatory reactions, growth promoters, cytokines, etc.; is involved in the production of PGE2; and is known to play an important role in IBD and autoimmune diseases [16]. NO expression and inflammatory cytokine production occur mainly through MAPKs [17]. JNK and ERK are among the downstream molecules of the MAPK pathway [18]. Activated ERK MAPK is required to induce the expression of iNOS and COX-2 and for the production of IL-6 and TNF-α in macrophages and microglia [19].

Regulating the local immune microenvironment is a key therapeutic goal. NSAIDs (e.g., aspirin, etoricoxib) and glucocorticoids (e.g., dexamethasone) are commonly used to relieve inflammation, but their long-term use can cause adverse effects such as gastrointestinal bleeding and osteoporosis, limiting their utility in disease management [20]. The induction of arthritis using Complete Freund’s Adjuvant (CFA) in mice provides a well-established platform for preclinical RA investigations [21].

The present study induced rheumatoid arthritis (RA) in mice via CFA injection, which led to joint swelling, synovial inflammation, and cartilage degradation. To evaluate the therapeutic potential of BTEX-K in this RA model, both in vivo micro-CT imaging and quantitative bone morphometric analyses were conducted. The effects of BTEX-K on the production of NO induced by LPSs in RAW 264.7, a macrophage, were investigated, and the effects on the signaling pathways were assessed.

## 2. Materials and Methods

### 2.1. Materials

The murine macrophage cell line RAW 264.7 was obtained from American Type Culture Collection (Manassas, VA, USA). Dulbecco’s modified Eagle’s medium (DMEM) was purchased from Sigma-Aldrich (St. Louis, MO, USA). Fetal bovine serum (FBS) was purchased from Access Biological (Vista, CA, USA). Penicillin–streptomycin and trypsin–EDTA solutions were purchased from Sigma-Aldrich (St. Louis, MO, USA). LPS was purchased from Sigma-Aldrich. IL-6 and TNF-α mouse ELISA kits were purchased from Elabscience Biotechnology Co., Ltd. (Wuhan, China). An ELISA kit for PGE2 was purchased from Cayman Chemical Company (Ann Arbor, MI, USA). Primary antibodies (iNOS, COX-2, ERK, P-ERK, JNK, P-JNK, IκBα, NF-κB, and β-actin) and the corresponding secondary antibodies for Western blotting analysis were purchased from Cell Signaling Technology (Danvers, MA, USA).

### 2.2. BTEX-K Production and HPLC Analysis

Dried red ginseng was extracted three times with 50% (*v*/*v*) ethanol in distilled water at 60 °C for 12 h (volume of 10 times). The extract was concentrated at 50 °C under reduced pressure. The crude extract (64 °Brix) was dissolved in 90% ethanol, and the supernatant was separated to obtain the second extract, which was then dissolved in water and combined with alpha-galactosidase (from *Aspergillus*) in 50 mM sodium acetate buffer (pH 4.5) at 60 °C for 96 h to obtain the final product (BTEX-K). The reactant was concentrated in powder form under vacuum conditions. The content of Compound K in BTEX-K was analyzed by High-Performance Liquid Chromatography (HPLC) using a 250 mm × 4.6 mm Thermo Hypersil Gold column (5 μm) with a flow of 1.6 to 2.5 mL min-1 in an agilent 1260 system (Agilent Technologies, Santa Clara, CA, USA) with a 10 μL loop valve. A variable-wavelength V4 absorbance detector set at 204 nm and a refractive index detector were used. The mobile phase consisted of varying concentrations of acetonitrile in water programmed by a system controller. All data was collected in triplicate at 30 °C with the potentiometric cell and columns thermostated with water jackets. The content of Compound K in BTEX-K is approximately 10% (102.5 mg/g).

### 2.3. Cell Culture

The RAW 264.7 cell line was purchased from American Type Culture Collection (Rockville, MD, USA). Cells were cultured in Dulbecco’s modified Eagle’s medium (DMEM, Sigma-Aldrich, St. Louis, MO, USA) containing 10% (*v*/*v*) fetal bovine serum, 100 U/mL penicillin, and 100 U/mL streptomycin at 37 °C in a humidified atmosphere of 95% air and 5% CO_2_.

### 2.4. Cell Viability Assay

The viability of RAW 264.7 cells in the presence of BTEX-K was measured using the MTT assay as previously described [22]. RAW 264.7 cells (1 × 10^5^ cells/mL) were seeded in 24-well plates for 24 h at 37 °C with 5% CO_2_. After 24 h, the medium was removed, and cells were treated with scaled concentrations (5, 10, 15, and 20 µg/mL) of BTEX-K for 24 h, after which each well was treated with MTT at 10% of the total volume at 37 °C for 1 h. MTT was aspirated, and the remaining formazan was dissolved in DMSO. Cell viability was measured at 595 nm using an FLx800 microplate reader (BioTek Instruments Inc., Winooski, VT, USA).

### 2.5. Nitric Oxide Assay

Nitric oxide was analyzed as previously described [23]. The RAW 264.7 macrophage cells were plated at a density of 1 × 10^5^ cells/mL in a 24-well plate for 24 h at 37 °C with 5% CO_2_. The culture medium was replaced with fresh medium containing various concentrations of BTEX-K (5, 10, 15, and 20 µg/mL) for 30 min prior to stimulation with LPS (1 µg/mL). After 18 h of incubation, the nitrite accumulation in the culture was measured as an indicator of NO production. For the assay, equal volumes of culture medium and Griess reagent were mixed, and the absorbance of each well was measured at 540 nm using an FLx800 microplate reader (BioTek Instruments Inc., Winooski, VT, USA). The culture medium was used as a blank for all experiments. The quantification of nitrite was standardized using NaNO_2_ at concentrations of 0 to 50 µM.

### 2.6. Measurement of Cytokine Levels

The levels of pro-inflammatory cytokines, namely IL-6, TNF-α, and PGE2 were measured in the cell culture media using ELISA kits as previously described [24]. RAW 264.7 macrophage cells were cultured in 6-well plates for 24 h at 37 °C and 5% CO_2_. The culture medium was replaced with fresh medium containing various concentrations of BTEX-K (5, 10, 15, and 20 µg/mL) for 30 min prior to stimulation with LPS (1 µg/mL). After 24 h of incubation, the concentration of IL-6, TNF-α, and PGE2 was measured using ELISA, according to the manufacturer’s instructions. A standard curve for each cytokine was constructed in parallel with the sample analysis.

### 2.7. Measurement of Hyaluronic Acid Level

Hyaluronic acid production was measured in a cell culture medium using an ELISA kit as previously described [25]. HaCaT cells were cultured at 37 °C and 5% CO_2_ on a 6-well plate for 24 h. After 24 h, the culture medium was replaced with a new medium containing various concentrations of BTEX-K (0, 5, 10, 15, and 20 μg/mL). After incubation for 24 h, the concentration of HA was measured using an ELISA kit according to the manufacturer’s instructions. The standard curve for each HA was constructed in parallel with the sample analysis.

### 2.8. Protein Extraction and Western Blotting

Western blotting was conducted as previously described [26]. RAW 264.7 cells were pretreated with various concentrations of BTEX-K for 30 min, then treated with 1 μg/mL LPS and cultured. The cells were lysed, and total protein concentration was determined using the Bradford reagent (Bio-Rad, Hercules, CA, USA). Proteins were resolved through the use of SDS–polyacrylamide gel electrophoresis (SDS-PAGE) and transferred to a polyvinylidene difluoride membrane. The membranes were incubated overnight at 4 °C with the respective primary antibodies (1:2500 dilution). The membranes were washed three times, and the peroxidase-conjugated antibody was used as the secondary antibody (1:5000 dilution). Proteins were detected using chemiluminescent plus detection reagent for Western blotting using the Image Quant LAS 4000 system (GE Healthcare, Buckinghamshire, UK).

### 2.9. Animal Experiment

The procedure for the induction of rheumatoid arthritis in animals was modified as previously described [27,28]. Female BALB/c mice were purchased from DBL Inc. (Eumseong, Republic of Korea). The animals were housed under controlled environmental conditions, including a temperature of 23 ± 3 °C, humidity of 45 ± 5%, and a 12 h light–dark cycle. They were given standard rodent chow (RodFeed; DBL Inc., Eumseong, Republic of Korea) and sterilized water ad libitum. After 1 week of adaptation, the mice were randomly divided into four groups (n = 5 per group), each with a normal control group (Control), a rheumatoid arthritis-induced group (RA), an RA Induction + 5 mg/kg BTEX-K administration group (RA5), and an RA Induction + 10 mg/kg BTEX-K administration group (RA10). The first step in rheumatoid arthritis animal production involved mixing a 2 mg/mL bovine type 2 collagen solution with the same concentration of complete Freund’s additive (CFA) at a ratio of 1:1 and slowly injecting 0.1 mL of the mixture solution through the skin below 1.5–3 cm at the base of the tail. Then, 1 week after the first induction, rheumatoid arthritis was induced by mixing the solution with incomplete Freund’s adjunct (IFA) instead of CFA and injecting it into one sole of the foot. BTEX-K administration was divided into the RA5 and RA10 groups administered doses at 5 mg/kg/day and 10 mg/kg/day, respectively. We euthanized the mice via cervical dislocation. All animal experiments were approved by the Yonsei University Institutional Animal Care and Use Committee (YWCI-202210-015-04) and carried out in accordance with the regulations.

### 2.10. Morphological Analysis of Articular Cartilage by Micro-CT

Micro-CT was performed as previously described [27,28]. In vivo micro-CT (Skyscan1176, Brucker, Karlsruhe, Germany) was used to photograph the foot of a small animal after the induction of rheumatoid arthritis (week 0) and 4 weeks after the experiment. Filming at week 0 was conducted the day after IFA drug injection to induce RA, and filming at week 4 was performed to determine the effectiveness of BTEX-K, which was conducted for a total of 4 weeks. Respiratory anesthesia was performed to minimize the movement of small animals when photographing a microtomography system in vivo (resolution: 18 μm; tube voltage: 65 kV; tube current: 278 μA; filter: 1.0 mm Al filter; exposure time: 520 ms; rotation step (deg): 0.7). The images obtained from in vivo micro-tumor systems were converted to two-dimensional cross-sectional gray-scale image slices via NRecon (Bruker micro CT, Kontich, Belgium). Bone mineral density (BMD), bone volume (BV), and crossectional thickness (Cs. Th) were analyzed. Based on the CT Analyzer (CT-AN, v1.10.9.0, Bruker micro-CT, Kontich, and Belgium) analysis results such as those obtained for trabecular thickness (Th). and objects per slice (Obj. N), the morphological changes in bone structure due to RA induction were measured.

### 2.11. Statistical Analysis

The experimental results are expressed as the mean ± the standard error of the mean (SEM). A one-way analysis of variance (ANOVA) followed by Dunn’s post hoc test and *t*-tests were performed. Statistical analyses were conducted using GraphPad Prsim 5 software. Statistical significance was indicated by *p*-values: * *p* < 0.05, ** *p* < 0.01, and *** *p* < 0.001.

## 3. Results

### 3.1. Effects of BTEX-K on LPS-Induced NO Production in RAW 264.7 Cells

**Figure 1:**
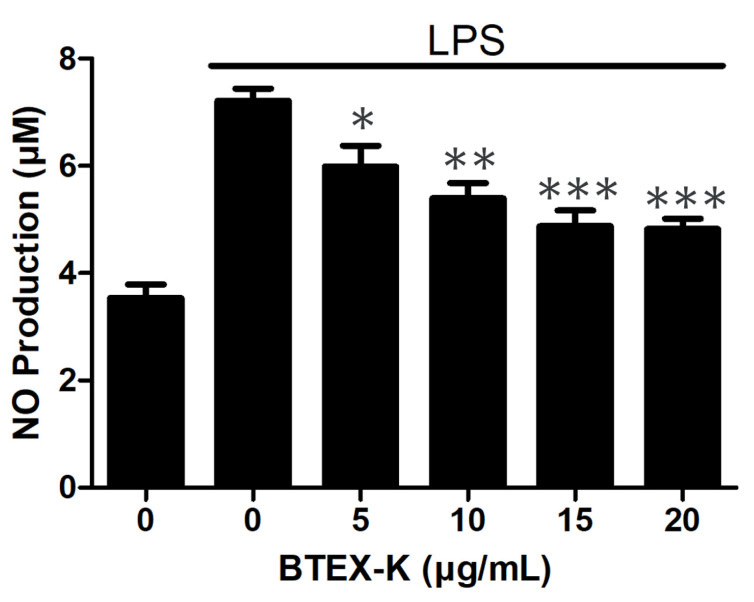
Effects of BTEX-K on LPS-induced NO production in RAW 264.7 cells. RAW 264.7 cells were pretreated with various concentrations of BTEX-K for 30 min and then stimulated with 1 µg/mL of LPS for 18 h. Medium supernatant was analyzed for nitrite using Griess reagent. Absorbance at 540 nm was measured using microplate reader. Data are representative of at least three independent experiments with similar results. Data are expressed as mean ± SEM (n = 4). * *p* < 0.05, ** *p* < 0.01, and *** *p* < 0.001 compared with positive control value stimulated by LPS alone.

To determine the effects of BTEX-K on inflammation in LPS-induced RAW 264.7 cells, the NO production of RAW 264.7 cells in response to varying BTEX-K concentrations was determined using NO assay. LPS treatment increased NO production in RAW 264.7 cells compared with that in untreated cells. However, BTEX-K significantly inhibited LPS-induced NO production in a concentration-dependent manner by 5.98 ± 0.39, 5.40 ± 0.29, 4.88 ± 0.29, and 4.82 ± 0.20 μM at the concentrations of 5, 10, 15, and 20 µg/mL, respectively (Figure 1).

### 3.2. Cytotoxicity of BTEX-K

**Figure 2:**
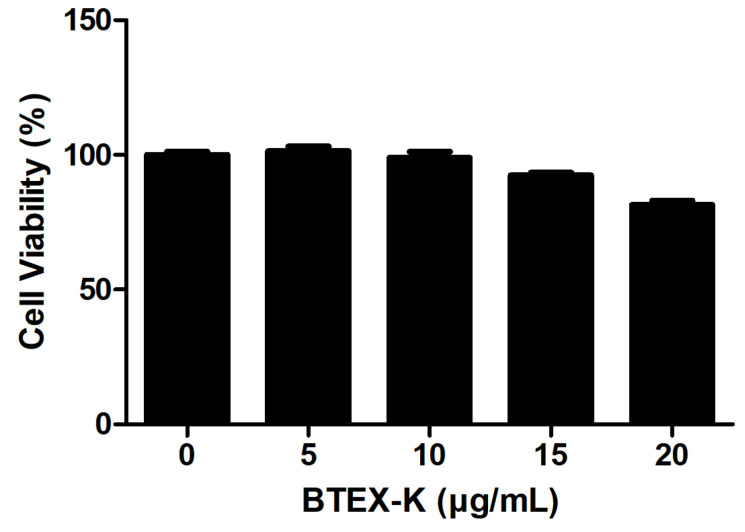
Effects of BTEX-K on cell viability in RAW 264.7 cells. RAW 264.7 cells were treated with BTEX-K (5, 10, 15, or 20 µg/mL) for 24 h. Cell viability was measured by MTT assay. Absorbance at 595 nm was measured using microplate reader. Data are representative of at least three independent experiments with similar results. Data are expressed as mean ± SEM (n = 4).

Cytotoxicity analysis was performed to determine the effects of BTEX-K on RAW 264.7 cells. RAW 264.7 cells were treated with BTEX-K at different concentrations for 24 h. As shown in Figure 2, BTEX-K was not cytotoxic. As a result of this, it was confirmed that the reduction in NO production is not due to any toxic effect of BTEX-K; rather, it is likely that BTEX-K exerts anti-inflammatory effects that inhibit the production of LPS-induced NO.

### 3.3. Effects of BTEX-K on Protein Expression of iNOS and COX-2 in LPS-Stimulated RAW 264.7 Cells

**Figure 3:**
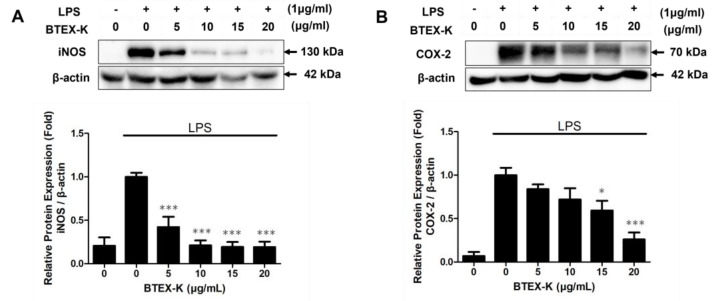
Effects of BTEX-K on protein expression of iNOS and COX-2 in LPS-stimulated RAW 264.7 cells. RAW 264.7 cells were pretreated with various concentrations of BTEX-K (0–20 µg/mL) for 30 min, followed by stimulation with LPS (1 µg/mL) for 24 h. (**A**) The expression of inducible nitric oxide synthase (iNOS) was determined by Western blotting. β-actin was used as a loading control. Representative blots and quantitative densitometric analysis of iNOS expression (normalized to β-actin) are shown. (**B**) The expression of cyclooxygenase-2 (COX-2) was measured by Western blotting. Protein expression was quantified using densitometry and normalized to β-actin. Data from triplicate experiments were quantified by densitometry. Data are expressed as mean ± SEM (n = 3). * *p* < 0.05 and *** *p* < 0.001 compared with positive control value stimulated by LPS alone.

BTEX-K inhibited NO production, which led us to further investigate the expression of iNOS and COX-2 proteins using Western blotting. As shown in Figure 3, LPS substantially increased the protein levels of iNOS and COX-2 after stimulation, whereas cells incubated with BTEX-K exhibited diminished protein levels. The relative densities were 0.42 ± 0.12, 0.21 ± 0.06, 0.19 ± 0.06, and 0.19 ± 0.06 fold for iNOS (Figure 3A) and 0.84 ± 0.05, 0.72 ± 0.13, 0.59 ± 0.11, and 0.26 ± 0.08 fold for COX-2 (Figure 3B) at the concentrations of 5, 10, 15, and 20 µg/mL, respectively. The results indicate that BTEX-K significantly inhibited iNOS and COX-2 protein levels in a dose-dependent manner.

### 3.4. Effects of BTEX-K on LPS-Induced Expression of Cytokines in RAW 264.7 Cells

**Figure 4:**
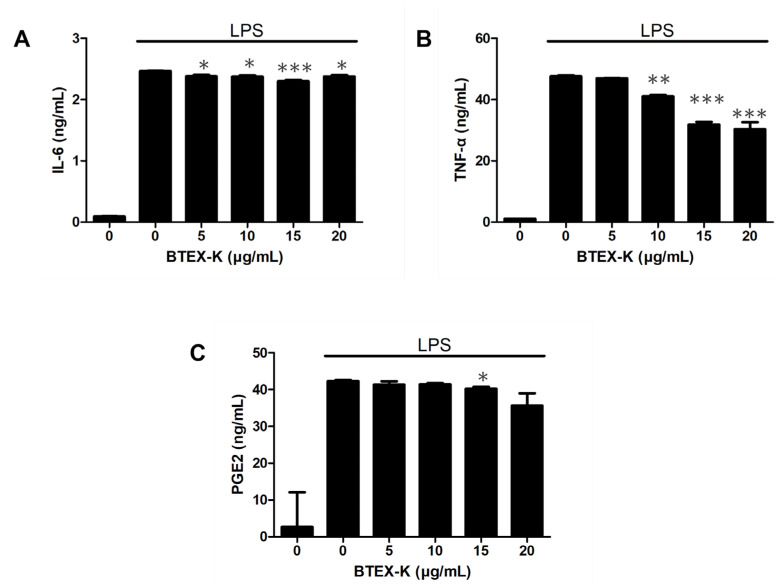
Effects of BTEX-K on LPS-induced expression of IL-6, TNF-α, and PGE2 in RAW 264.7 cells. RAW 264.7 cells were pretreated with various concentrations of BTEX-K (0–20 µg/mL) for 30 min, followed by stimulation with LPS (1 µg/mL) for 24 h. (**A**) The concentration of IL-6 in the culture supernatant was measured using an ELISA kit. (**B**) The production of TNF-α was quantified using ELISA. (**C**) The secretion level of prostaglandin E2 (PGE2) in the culture supernatant was quantified using a commercial ELISA kit after 24 h. Data are representative of at least three independent experiments with similar results. Data are expressed as mean ± SEM (n = 3). * *p* < 0.05, ** *p* < 0.01, and *** *p* < 0.001 compared with positive control value stimulated by LPS alone.

The levels of pro-inflammatory cytokines, IL-6, TNF-α, and PGE2, play important roles in mediating inflammatory disease. The levels of IL-6, TNF-α, and PGE2 in RAW 264.7 cell line culture supernatants after LPS stimulation with and without BTEX-K were examined using ELISA. Figure 4 shows that BTEX-K dose-dependently inhibited the levels of IL-6, TNF-α, and PGE2 in the cell supernatant. Thus, BTEX-K inhibits the LPS-stimulated levels of IL-6, TNF-α, and PGE2 in macrophages.

### 3.5. Effects of BTEX-K on Hyaluronic Acid Expression in HaCaT Cells

**Figure 5:**
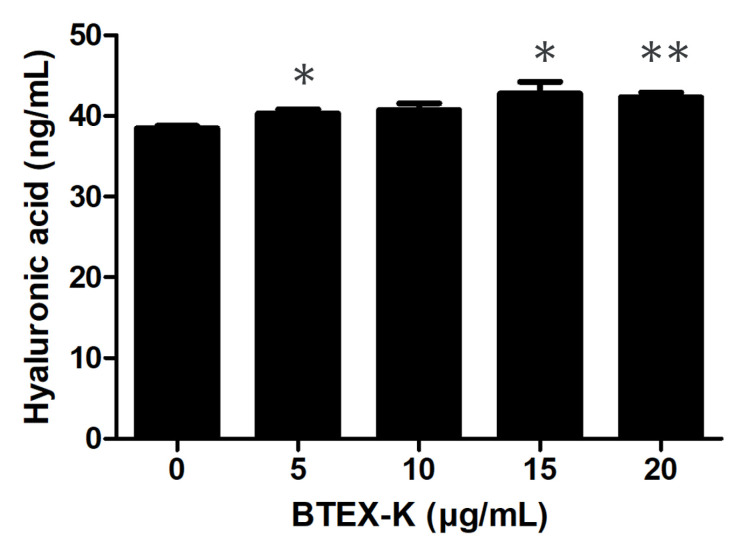
Effects of BTEX-K on expression of HA in HaCaT cells. Cells were cultured for 24 h and treated with various concentrations of BTEX-K for 24 h. After 24 h, level of HA in cultured supernatant was measured using ELISA kit. Data are representative of at least three independent experiments with similar results. Data are expressed as mean ± SEM (n = 3). * *p* < 0.05 and ** *p* < 0.01 compared with BTEX-K untreated group.

The level of HA in the HaCaT cell line culture supernatant with or without BTEX-K was investigated using ELISA. Figure 5 shows that BTEX-K increases the production of HA in the cell supernatant. As can be seen in Figure 5, the produced amount of HA was significantly increased by BTEX-K. The amounts were 40.30 ± 0.52, 40.73 ± 0.83, 42.76 ± 1.45, and 42.33 ± 0.57 ng/mL at the concentrations of 5, 10, 15, and 20 µg/mL, respectively.

### 3.6. Effects of BTEX-K on ERK and JNK Phosphorylation in LPS-Stimulated RAW 264.7 Cells

**Figure 6:**
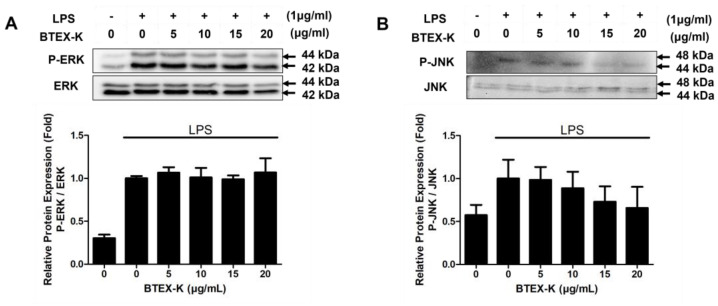
Effects of BTEX-K on JNK and ERK phosphorylation in LPS-stimulated RAW 264.7 cells. RAW 264.7 cells were pretreated with various concentrations of BTEX-K (0–20 µg/mL) for 30 min and then stimulated with LPS (1 µg/mL) for 30 min. (**A**) Total cell lysates were subjected to Western blotting to assess the expression levels of phosphorylated ERK (P-ERK) and total ERK. Relative protein levels were quantified by densitometric analysis and normalized to total ERK. (**B**) The levels of phosphorylated JNK (P-JNK) and total JNK were measured by Western blotting. Densitometric analysis was performed and the results were normalized to total JNK. Data from triplicate experiments were quantified by densitometry. Data are expressed as mean ± SEM (n = 3).

MAPK pathways play an important role in managing various physiological processes, regulating the production of inflammatory cytokines as well as the expression of inflammatory mediators, such as iNOS and COX-2. We examined the effects of BTEX-K on the MAPK signaling pathway after LPS stimulation by Western blotting. After inducing the RAW 264.7 cells with LPS (1 µg/mL) for 30 min, the phosphorylation levels of ERK increased significantly (1 ± 0.03). Treatment with BTEX-K ineffectively reduced the phosphorylation levels of ERK (Figure 6A). As shown in Figure 6B, the phosphorylation levels in the treatment of RAW 264.7 cells with LPS alone were increased (1 ± 0.22), whereas the phosphorylation levels of JNK were inhibited by BTEX-K. The relative densities were 0.98 ± 0.15, 0.89 ± 0.19, 0.73 ± 0.18, and 0.66 ± 0.25 fold at the concentrations of 5, 10, 15, and 20 µg/mL, respectively. All these results indicate that BTEX-K regulates the MAPK signaling pathway by inhibiting the phosphorylation of JNK.

### 3.7. Effects of BTEX-K on NF-κB Signaling Pathway in LPS-Induced RAW 264.7 Cells

**Figure 7:**
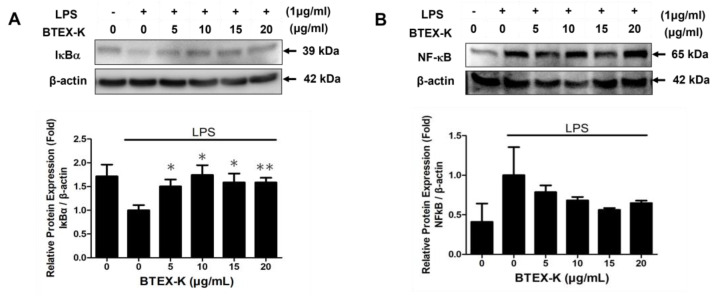
Effects of BTEX-K on NF-κB signaling pathway in LPS-induced RAW 264.7 cells. (**A**) RAW 264.7 cells were pretreated with various concentrations of BTEX-K (0–20 µg/mL) for 30 min and then stimulated with LPS (1 µg/mL) for 30 min. Total cell lysates were analyzed by Western blotting to determine the expression levels of IκBα. Relative levels were quantified by densitometry and protein expression was normalized to β-actin. (**B**) Under the same pretreatment conditions, cells were stimulated with LPS for 6 h, and the expression of NF-κB was analyzed via Western blotting. Densitometric quantification was performed and normalized to β-actin. Data are expressed as mean ± SEM (n = 4). * *p* < 0.05 and ** *p* < 0.01 compared with positive control value stimulated by LPS alone.

The activation of NF-κB plays important roles in the LPS-induced expression of inflammatory mediators and cytokines such as iNOS, COX-2, IL-6, TNF-α, and PGE2 in RAW 264.7 cells. NF-κB is a complex that is kept inactive in the cytoplasm by IκBα. When LPS activates the transcription factor NF-κB, IκBα is phosphorylated and degraded, and the NF-κB separated from IκB is translocated into the nucleus, thus playing a role as an inflammatory transcription factor. To identify the inhibitory mechanism of BTEX-K, its effect on IκBα degradation and on the NF-κB expression level were measured via Western blot. As shown in Figure 7A, the group treated with only LPS showed a decrease in IκBα (1 ± 0.11). However, in the group treated with BTEX-K, it was confirmed that the decrease in IκBα was significantly suppressed by BTEX-K. The relative densities were 1.50 ± 0.15, 1.74 ± 0.21, 1.59 ± 0.19, and 1.58 ± 0.10 fold at the concentrations of 5, 10, 15, and 20 µg/mL, respectively. We also measured the protein level of NF-κB by Western blotting. As shown in Figure 7B, BTEX-K suppressed the protein level of NF-κB. All these results suggest that BTEX-K reduced LPS-induced NF-κB activation.

### 3.8. Effects of BTEX-K on Protein Expression of PPAR-γ in LPS-Stimulated RAW 264.7 Cells

**Figure 8:**
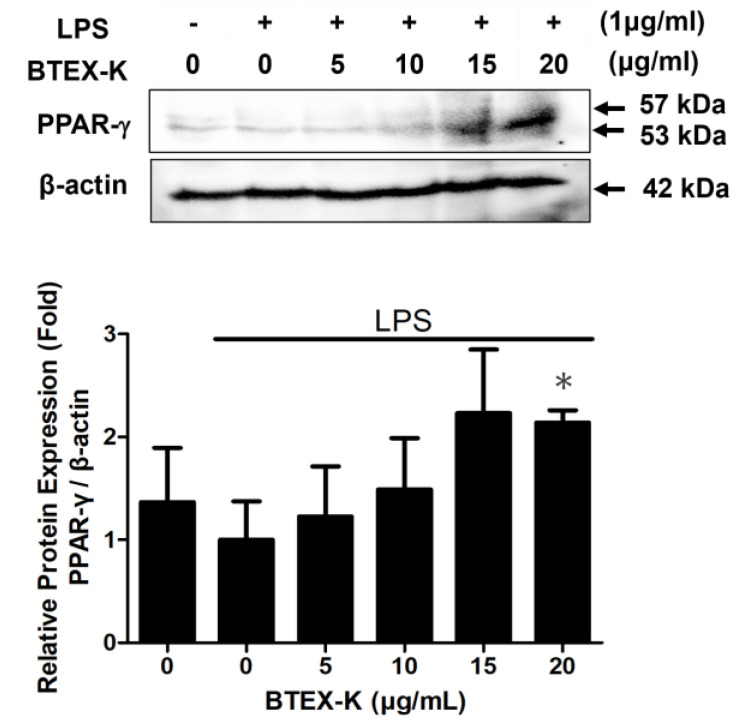
Effects of BTEX-K on protein expression of PPAR-γ in LPS-induced RAW 264.7 cells. Cells were pretreated with BTEX-K for 30 min prior to stimulation with LPS for 18 h. Total cell lysates were extracted and examined via Western blotting. Data from triplicate experiments were quantified by densitometry. Data are expressed as mean ± SEM (n = 4). * *p* < 0.05 compared with positive control value stimulated by LPS alone.

PPAR-γ demonstrates anti-inflammatory effects by regulating the NF-κB signal pathway. To further identify the mechanism underlying the anti-inflammatory effects of BTEX-K, the degradation of the PPAR-γ protein was examined in LPS-induced RAW 264.7 cells exposed to BTEX-K. When induced with only LPS, the PPAR-γ protein was degraded. However, the degradation of the PPAR-γ protein was significantly inhibited by BTEX-K. The relative densities were 1.23 ± 0.49, 1.49 ± 0.50, 2.23 ± 0.62, and 2.23 ± 0.62 fold at the concentrations of 5, 10, 15, and 20 µg/mL, respectively (Figure 8). These results suggest that BTEX-K could enhance PPAR-γ transcriptional activity in mononuclear macrophages.

### 3.9. Effects of BTEX-K on Bone Structure and Density in Rheumatoid Arthritis-Induced Mice Assessed by In Vivo Micro-CT

**Figure 9:**
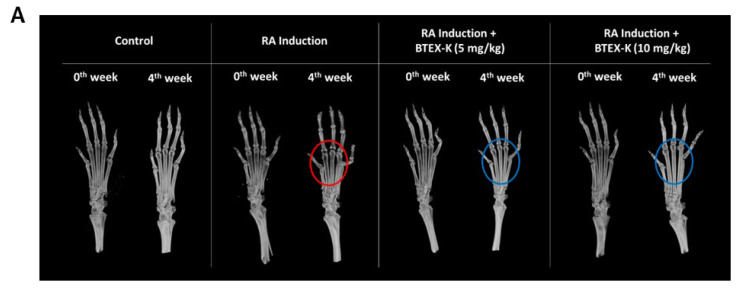

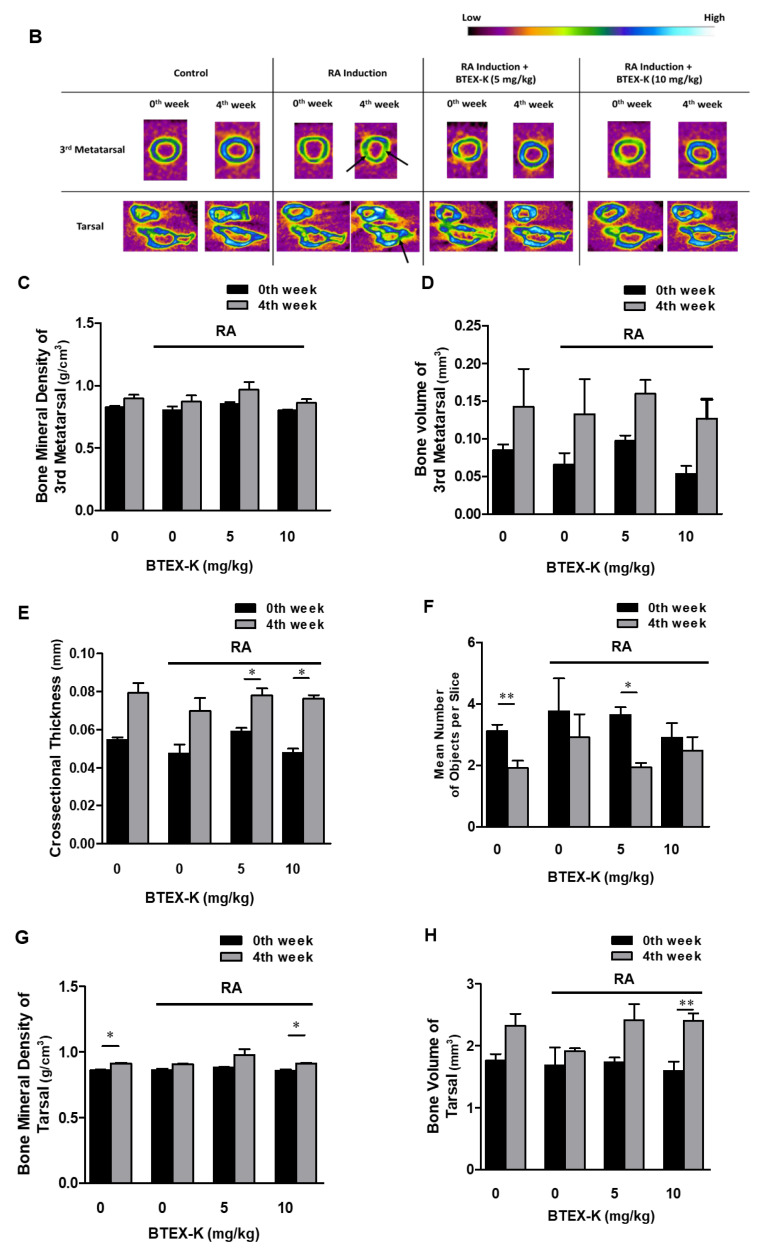

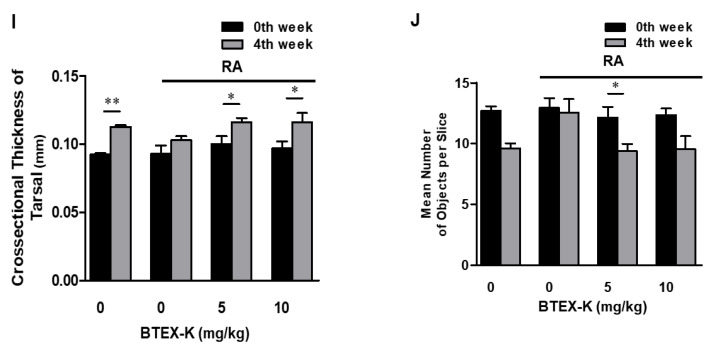
Morphological analysis of articular cartilage by micro-CT. (**A**) For micro-CT image analysis of hind foot in rheumatoid arthritis model of BALB/c mice, 3D images of mouse foot tissue before and after experiment are shown. After 4 weeks, intensive cancellous bone weakness (red circle) was seen in the metatarsal area in RA-induced subjects. However, both BTEX-K treatment groups showed higher bone mineral density and bone volume (blue circle) compared to the RA-induced group. (**B**) In rheumatoid arthritis model of BALB/c mice, cross-sectional images of 3rd metatarsal and tarsal sections before and after experiment were identified as two-dimensional gray-scale image slices through images obtained from in vivo microtidal systems for bone color analysis (**B**). Comparison of (**C**) bone mineral density, (**D**) bone volume, (**E**) Crossectional Thickness of Tarsal, and (**F**) Mean Number of Objects per Slice values for week 4 versus week 0 of each group with respect to 3rd metatarsal in rheumatoid arthritis model of BALB/c mice. Comparison of (**G**) bone mineral density, (**H**) bone volume, (**I**) Crossectional Thickness of Tarsal, and (**J**) Mean Number of Objects per Slice values for week 4 versus week 0 of each group with respect to tarsal bones in rheumatoid arthritis model of BALB/c mice. Data are expressed as mean ± SEM (n = 5). * *p* < 0.05 and ** *p* < 0.01 compared with week 0 for each group.

Photographs at week 0 after the induction of rheumatoid arthritis were taken the day after IFA drug injection for RA induction, and photographs at week 4 were taken to understand the morphological changes in BTEX-K over a total of 4 weeks. After 4 weeks, intensive cancellous bone weakness (red circle) was seen in the metatarsal area in RA-induced subjects. However, both BTEX-K treatment groups showed higher bone mineral density and bone volume (blue circle) compared to the RA-induced group (Figure 9A).

Through the images obtained from the in vivo microtomography system, a two-dimensional cross-sectional gray-scale image slice was confirmed. According to the scale bar at the top of Figure 9B, the higher the bone density, the closer to the High color, and the lower the bone density, the closer to the Low color. Looking at the images of the RA-induced group after 4 weeks, it can be seen that the cortical bone was noticeably weakened (*Arrow*) in the third metatarsal due to rheumatoid arthritis, but all of the RA-induced + BTEX-K treatment groups showed similar results to those of the control without arthritis. Quantitative and qualitative conditions of bone were shown. This can be interpreted as meaning that BTEX-K can suppress the state of the cortical bone weakened by arthritis. In the case of the tarsal bones, it was found that the bone connection state was significantly lowered (*Arrow*) compared to other groups due to rheumatoid arthritis. In the RA-induced + BTEX-K treatment groups, connectivity was greatly restored, suggesting that BTEX-K can improve the connectivity of bone tissue weakened by arthritis (Figure 9B).

BMD was measured before and after the experiment to measure the amount of bone mineral in bone tissue following the induction of arthritis and the oral administration of BTEX-K. There was no significant difference in BMD regardless of whether arthritis was induced or not and whether BTEX-K was administered (Figure 9C). In Figure 9D, BV is the calculated value of bone volume, and bone volume tended to increase in all groups. However, there was no significant difference in bone volume according to whether arthritis was induced or not and whether BTEX-K was administered. Cross-sectional thickness is an index that refers to the average thickness of a bone cross-section, and no significant change was seen depending on whether or not arthritis was induced. However, cross-sectional thickness was significantly increased in all BTEX-K treatment groups, indicating that the BTEX-K drug is effective in increasing cortical bone thickness (Figure 9E). The mean number of objects per slice refers to the average number of bone segments in cross-sectional images, and when arthritis is induced, the number of segments increases due to a decrease in bone connectivity. Therefore, it was determined that the lower the number of segments, the better the bone condition morphologically. Figure 9F shows that a significant decrease was seen in this value for the control in which arthritis was not induced, and no significant change was seen in the RA induction group. As for the groups with RA induction and administered BTEX-K, only the 5 mg/kg concentration group showed a significant decrease. This suggests that BTEX-K can inhibit the deterioration of bone connectivity that can occur due to arthritis.

As shown in Figure 9G, there was a significant increase in bone mineral density in the control group, but no significant change was shown in the RA-induced group according to the induction of arthritis. Bone mineral density showed a tendency to increase in all RA-induced + BTEX-K administration groups, but it was significantly increased in the 10 mg/kg concentration group. This suggests that BTEX-K can inhibit bone mineral density which can be lowered due to arthritis. In Figure 9H, BV increased to about 1.33 times in the control but increased to 1.17 in the RA-induced group, so it seems that the increase in bone volume decreased according to the induction of arthritis, but there was no significant change within the group. In the RA induction and BTEX-K administration groups, the increase was about 1.38 times at 5 mg/kg and about 1.52 times at 10 mg/kg. However, a significant increase was shown only in the 10 mg/kg treatment group. This suggests that BTEX-K can inhibit the decrease in bone volume that can occur due to arthritis. As seen in Figure 9I, no significant change was shown in the RA-induced group, but bone volume did increase significantly in the control group. This means that rheumatoid arthritis can affect bone thickness. Considering that cross-sectional thickness was significantly increased in all BTEX-K treatment groups, it is determined that the BTEX-K drug is effective in increasing cortical bone thickness. In Figure 9J, cortical bone thickness showed a tendency to decrease in the control without arthritis, but relatively no change in the RA-induced group was seen. In the RA induction and BTEX-K-administered groups, the 5 mg/kg concentration group and the 10 mg/kg concentration group showed a significant decrease. This suggests that BTEX-K can inhibit the deterioration of bone connectivity that can occur due to arthritis.

## 4. Discussion

In this study, an inflammatory response was induced in RAW 264.7 cells by exposing them to LPS, and the effects of BTEX-K treatment were assessed. The goal of this study was to investigate the potential anti-inflammatory effects of BTEX-K, with the larger goal of alleviating chronic inflammatory response in various diseases. Inflammation is a complex process mediated by activated inflammatory cells of the immune system, including macrophages [29]. However, chronic inflammation causes various chronic diseases, such as arthritis, autoimmune diseases, cancer, cardiovascular diseases, Alzheimer’s disease, neurological diseases, lung diseases, and type 2 diabetes [30]. LPS, derived from Gram-negative bacteria, stimulates macrophages to produce the potential vasodilator NO and a series of pro-inflammatory mediators including the cytokines IL-6 and TNF-α [31].

In the present study, we confirmed that BTEX-K reduces the production of NO, an inflammatory marker, in macrophages induced by LPS. When BTEX-K was added at 5, 10, 15, and 20 µg/mL, the amount of LPS-induced NO was reduced in a concentration-dependent manner (Figure 1). In addition, a cytotoxicity test was performed; the results showed that the decrease in NO production by BTEX-K due to LPS was not a result of cytotoxicity (Figure 2). NO is involved in cell-mediated immune response, blood vessel relaxation, and neurotransmission; when macrophages are stimulated with LPS, iNOS is expressed to produce NO [32]. NO is a substance that mediates the inflammatory response, and activated macrophages produce pro-inflammatory cytokines such as IL-6 and TNF-α [33]. The excessive production of inflammatory mediators causes chronic inflammation to worsen various human diseases. The results of our study confirmed that BTEX-K inhibited NO production (Figure 1). iNOS is a synthetase of NO that increases expression in LPS-induced macrophages and directly induces the production of NO [34]. It was confirmed that BTEX-K reduced the protein expression and production of iNOS, thus showing that it was directly involved in suppressing the production of NO (Figure 3A). The effects of BTEX-K on the protein expression of COX-2, which mediates the production of PGE2, were also investigated [35]. Similarly to iNOS, COX-2 protein expression was suppressed depending on the treatment concentration of BTEX-K (Figure 3B).

We confirmed that BTEX-K decreased the production of the pro-inflammatory cytokines IL-6 and TNF-α (Figure 4A,B). In addition, PGE2, like NO, is synthesized by COX-2, which is an important inflammatory mediator mainly involved in the transmission of pain and heat to damaged areas or tissues and induces an excessive immune response that causes various inflammatory diseases, such as MS and Parkinson’s disease. When LPS-induced macrophages were treated with BTEX-K, the production of PGE2 was inhibited (Figure 4C), similarly to a previous report [36]. Based on the above results, BTEX-K was thought to have anti-inflammatory effects; therefore, a molecular study was conducted. HA is a component of cartilage tissue that helps the binding of molecules in cartilage tissue and strengthens cartilage to withstand joint movement and weight. The decrease in HA components in cartilage and synovial fluid is one of the causes of osteoarthritis, which reduces the absorption of shock in the joints and stiffens the joints, making walking uncomfortable. When BTEX-K was treated with HaCaT cells, it was confirmed that the amount of HA produced increased (Figure 5). This means that BTEX-K has the effect of reinforcing cartilage.

LPS regulates iNOS and COX-2 expression via the MAPK signaling pathway that includes the phosphorylation of p38, ERK, and JNK, and its activation induces NF-κB activation in macrophages. The activation of MAPK has been demonstrated to be important for the regulation of iNOS and COX-2 expression through the activation of NF-κB [37]. First, we confirmed the effects of BTEX-K by examining the degree of phosphorylation of JNK and ERK, which are molecules that regulate the activation of transcription factors in the MAPK pathway [38]. BTEX-K did not reduce the LPS-induced phosphorylation of ERK (Figure 6A) but decreased the LPS-induced phosphorylation of JNK (Figure 6B). This made it possible to anticipate that the activation of transcription factors in the MAPK pathway would be inhibited. The transcription factor NF-κB plays an important role in immune and inflammatory responses [39]. In most cell types, NF-κB dimers are retained in the cytoplasm through interactions with IκB inhibitory proteins [40]. Separation by the phosphorylation of IκBα leads to a chronic inflammation-related disease due to the activation of NF-κB. Therefore, to check whether BTEX-K is effective in inflammatory reactions, LPS-induced macrophages were treated with BTEX-K, the degree of degradation of IκBα was observed, and IκB recovery by BTEX-K treatment was confirmed (Figure 7A). We also confirmed that BTEX-K inhibited NF-κB activation (Figure 7B). PPAR-γ acts as a trans-repressor of macrophage inflammatory genes [41]. We examined whether BTEX-K increased the expression of PPAR-γ. BTEX-K increased the level of PPAR-γ expression (Figure 8), and these results suggest that the activation of NF-KB is suppressed by increasing PPAR-γ expression. Taken together, our findings confirmed that BTEX-K inhibits the protein expression of iNOS, the main mediator of inflammatory diseases, and inhibits the expression of inflammatory cytokines through the direct inhibition of inflammation-related signaling pathways, the NF-κB pathway and the MAPK pathway.

We measured the morphological changes in bone structure due to RA induction by analyzing bone density, bone volume, cross-section thickness, and bone segmentation by photographing the sole area of small animals after rheumatoid arthritis induction (week 0) and 4 weeks after the experiment using an in vivo microtome system. As a result, the BTEX-K treatment group showed higher bone density and bone mass compared to the RA-induced group. In addition, although rheumatoid arthritis significantly weakened the cortical bone in the third metatarsal, both RA-induced + BTEX-K treatment groups showed a quantitative and qualitative state of bone similar to that of the control, which means that BTEX-K can inhibit the condition of the cortical bone weakened by arthritis. In the case of the tarsal bones, it was confirmed that the connection state of the bone was significantly lower than that of other groups due to rheumatoid arthritis. In the RA-induced + BTEX-K treatment groups, it was confirmed that connectivity recovered significantly, indicating that BTEX-K can improve the connectivity of bone tissue weakened by arthritis (Figure 9). From the above results, it is determined that BTEX-K exhibits an arthritis-relieving effect via the anti-inflammatory effect that inhibits macrophage activation.

BTEX-K is a red ginseng extract with enhanced Compound K levels through fermentation in the manufacturing process of conventional red ginseng extract. The main component of BTEX-K is Compound K, which is different from that of conventional raw materials (ginseng and red ginseng). Compound K is known to be produced in ginsenoside Rb1 and Rb2 Rc through bioconversion by the β-glucosidase (glucosidase) of human intestinal microorganisms [42]. Fermented/enzyme-treated red ginseng extract facilitates the absorption of ginsenoside in the human body [43].

We conclude that BTEX-K exhibits excellent anti-inflammatory activity and, thus, has potential as a rheumatoid arthritis agent. However, in this study, only partial mechanisms of the NF-κB and MAPK signaling pathways were examined, and the receptor-level actions and upstream regulators of BTEX-K were not identified. In addition, the long-term safety, pharmacokinetics, and in vivo efficacy of BTEX-K have not yet been evaluated through animal or clinical studies, which limits the clinical applicability of the findings.

## 5. Conclusions

**Figure 10:**
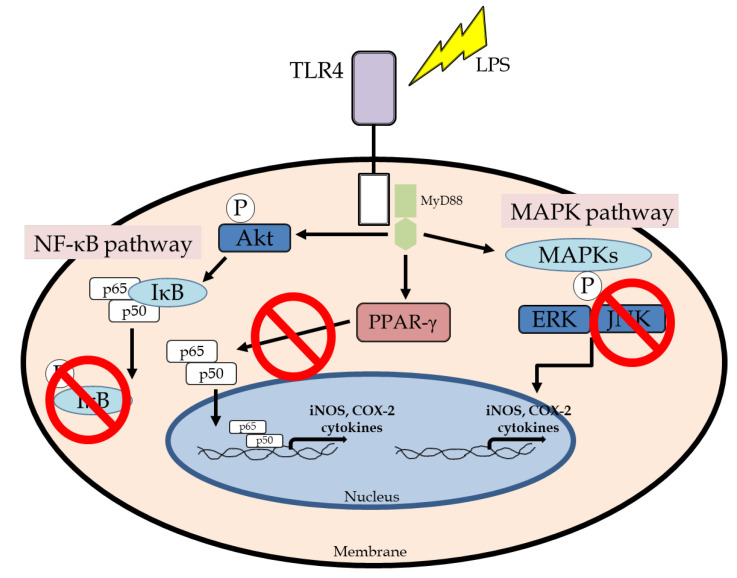
BTEX-K inhibiting inflammatory response via MAPK and NF-κB pathways in LPS-induced macrophages.

In this study, our results demonstrated the anti-inflammatory effects of BTEX-K, which is dried red ginseng combined with alpha galactosidase, on the activation of RAW 264.7 macrophages. The production of NO in BTEX-K-treated groups was significantly decreased as compared to the LPS group. BTEX-K inhibited the levels of pro-inflammatory cytokines IL-6, TNF-α, and PGE2 in LPS-induced RAW 264.7 cells. BTEX-K also reduced the phosphorylation levels of JNK through the MAPK pathway and inhibited NF-κB activation (Figure 10). Thus, we suggest that BTEX-K can strongly inhibit the inflammatory response to LPS in macrophages.

## Data Availability

The data that support the findings of this study are available from the corresponding author upon reasonable request.
